# Draft Genome Sequences of Bacillus megaterium KU143, Microbacterium testaceum KU313, and Pseudomonas protegens AS15, Isolated from Stored Rice Grains

**DOI:** 10.1128/genomeA.00468-18

**Published:** 2018-05-31

**Authors:** Jin-Ju Jeong, Hyeon-Ji Moon, Duleepa Pathiraja, Byeonghyeok Park, In-Geol Choi, Ki Deok Kim

**Affiliations:** a Laboratory of Plant Disease and Biocontrol, Department of Biosystems and Biotechnology, Korea University, Seoul, South Korea; b Department of Biotechnology, Korea University, Seoul, South Korea

## Abstract

Bacillus megaterium KU143, Microbacterium testaceum KU313, and Pseudomonas protegens AS15 from stored rice grains exhibited antifungal activity against Aspergillus and Penicillium spp. predominant in stored rice. Here, we report their bacterial draft genomes, which contain genes related to biotic and abiotic stress management, as well as antimicrobial and insecticidal traits.

## GENOME ANNOUNCEMENT

Previously, 460 bacterial strains were isolated from stored rice grains obtained from rice-processing complexes from 11 different regions in South Korea (1, 2). Among them, Bacillus megaterium KU143, Microbacterium testaceum KU313, and Pseudomonas protegens AS15 were selected as potential biocontrol agents (3–5). These bacterial strains showed significant biocontrol activity against Aspergillus flavus growth and aflatoxin production as well as the predominant fungi (A. candidus, A. fumigatus, Penicillium fellutanum, and Penicillium islandicum) in stored rice grains (3–5). Furthermore, B. megaterium KU143 and P. protegens AS15 produced volatile compounds that could reduce conidial germination and germ tube elongation, as well as mycelial growth of the predominant fungi (4, 5). Here, we present the draft genome sequences of these strains, KU143, KU313, and AS15, belonging to different genera and exhibiting significant biocontrol activity against rice fungal contamination.

Genome sequencing of bacterial strains KU143, KU313, and AS15 was performed using the Illumina MiSeq platform at the Computational and Synthetic Biology Laboratory, Korea University (Seoul, South Korea). Totals of 336,941, 955,850, and 531,468 paired-end reads (40.3-, 158.3-, and 47.2-fold coverage) for KU143, KU313, and AS15, respectively, were generated from paired-end sequencing of the genomic library with an average insert size of 500 bp. Low-quality reads were trimmed with a quality threshold of Q20, and the trimmed reads were subjected to *de novo* assembly using the SPAdes assembler (6). The reads were assembled to 98, 79, and 52 scaffolds for KU143, KU313, and AS15, respectively, with total lengths and G+C contents as shown in Table 1. The maximum lengths and *N*_50_ values of the contigs were 542,591 and 192,911 bp for KU143, 768,312 and 756,039 bp for KU313, and 1,020,915 and 290,736 bp for AS15, respectively. The genomes were annotated with the NCBI Prokaryotic Genome Annotation Pipeline (PGAP). The 4,995, 3,298, and 6,040 coding sequences of KU143, KU313, and AS15 showed 48.94, 29.46, and 60.18% sequence similarities to known genes in the NCBI database, respectively. In addition, retrieved numbers of tRNA, 5S rRNA, 16S rRNA, and 23S rRNA sequences of the strains are shown in Table 1.

**TABLE 1:** Summary of genome sequencing and GenBank accession and version numbers of B. megaterium KU143, M. testaceum KU313, and P. protegens AS15

Bacterial strain	Genome size (bp)	G+C content (%)	No. of coding sequences	No. of tRNAs	No. of rRNAs			GenBank accession no.	Version no.
					5S	16S	23S		
B. megaterium KU143	5,022,643	38.04	4,995	102	15	22	20	POTF00000000	POTF01000000
M. testaceum KU313	3,623,185	69.51	3,298	46	4	1	2	PPEE00000000	PPEE01000000
P. protegens AS15	6,756,833	63.52	6,040	60	7	1	1	PPEF00000000	PPEF01000000

In general, the genomes of B. megaterium KU143, M. testaceum KU313, and P. protegens AS15 contain genes related to biocontrol traits (i.e., siderophore, polyketide, phosphate solubilization, motility, and biofilm formation) that may be significant factors in the host colonization and protection against pathogenic infections (7–10). Additionally, biotic and abiotic stress management genes (i.e., superoxide dismutase and catalase) were found in all the examined bacterial strains (11, 12). In particular, the genome of strain AS15 has several genes related to antimicrobial or insecticidal compounds (i.e., hydrogen cyanide synthase, ATP-dependent zinc metalloprotease, and chitinase) (13–15). Therefore, the genomes of these bacterial strains belonging to different genera may help to increase our understanding of the characteristics of their biocontrol of fungal contamination in stored rice grains.

### Accession number(s).

This whole-genome shotgun project has been deposited in DDBJ/EMBL/GenBank under the accession and version numbers listed in Table 1.
